# Overexpression of the secretory small GTPase Rab27B in human breast cancer correlates closely with lymph node metastasis and predicts poor prognosis

**DOI:** 10.1186/1479-5876-10-242

**Published:** 2012-12-05

**Authors:** Jia-Xing Zhang, Xiao-Xia Huang, Man-Bo Cai, Zhu-Ting Tong, Jie-Wei Chen, Dong Qian, Yi-Ji Liao, Hai-Xia Deng, Ding-Zhun Liao, Ma-Yan Huang, Yi-Xin Zeng, Dan Xie, Shi-Juan Mai

**Affiliations:** 1 State Key Laboratory of Oncology in South China, Cancer Center, Sun Yat-Sen University, 651# Dongfeng Road East, Guangzhou, 510060, China; 2 Department of Pathology, Cancer Center, Sun Yat-Sen University, 651# Dongfeng Road East, Guangzhou, 510060, China; 3 Cancer Center, The First Affiliated Hospital, University of South China, Hengyang, China; 4 Department of Radiotherapy, The First Affiliated Hospital, Anhui Medical University, Hefei, China

**Keywords:** Rab27B, Breast cancer, Prognosis, EMT, Metastasis

## Abstract

**Background:**

The secretory small GTPase Rab27b was recently identified as an oncogene in breast cancer (BC) in vivo and in vitro studies. This research was designed to further explore the clinical and prognostic significance of Rab27B in BC patients.

**Methods:**

The mRNA/protein expression level of Rab27B was examined by performing Real-time PCR, western blot, and immunohistochemistry (IHC) assays in 12 paired BC tissues and matched adjacent noncancerous tissues (NAT). Then we carried out IHC assay in a large cohort of 221 invasive BC tissues, 22 normal breast tissues, 40 fibroadenoma (FA), 30 ductual carcinoma in situ (DCIS) and 40 metastatic lymph nodes (LNs). The receiver operating characteristic curve method was applied to obtain the optimal cutoff value for high Rab27B expression. Epithelial-mesenchymal transition (EMT) marker expression levels were detected in relation to Rab27B expression.

**Results:**

We observed that the increased expression of Rab27B was dependent upon the magnitude of cancer progression (*P* < 0.001). The elevated expression of Rab27B was closely correlated with lymph node metastasis, advanced clinical stage, ascending pathology classification, and positive ER status. Furthermore, patients with high expression of Rab27B had inferior survival outcomes. Multivariate Cox regression analysis proved that Rab27B was a significantly independent risk factor for patients’ survival (*P* < 0.001). Furthermore, a significant positive relationship was observed between Rab27B expression and elevated mesenchymal EMT markers.

**Conclusion:**

Our findings suggest that overexpression of Rab27B in BC coincides with lymph node metastasis and acquisition of a poor prognostic phenotype.

## Background

Breast cancer (BC) is by far the leading malignancy in women and is a serious threat to the health of women worldwide [1-3]. Despite the great advance achieved in therapy technology recently, the prognosis of the cancer remains unsatisfactory in the clinic [4]. Substantial effort has focused on the gene alterations and specific molecular markers that are responsible for the tumorigenesis and progression of this malignancy. Because of prognostic markers, including estrogen receptor (ER), progesterone receptor (PR), CerbB-2, carcinoembryonic antigen, CA 15–3, CA 27.29, the risk classification of a BC patient’s outcome can be defined more accurately. Thus, identification of more biomarkers that could be used to define the progression of this malignancy may be of great benefit to BC patients [5]. However, the identification of promising molecular biomarkers in BC that have clinical significance is still substantially limited.

Rab GTPases, which function as molecular switches that alternate between active GTP-bound and inactive GDP-bound conformational states, constitute the largest family of small GTPases and play a vital role in endocytosis and exocytosis vesicle-trafficking control [6-9]. When Rabs are activated, the vesicles are engaged to specific effectors required for vesicle movement, docking, and fusion [10]. The secretory Rab GTPases, including Rab26, Rab37, Rab3A/B/C/D, and Rab27A/B, are reported to be responsible for regulated vesicle exocytosis [6]. The Rab27 subfamily, including the homologues Rab27A and B, are 71% identical at the amino acid level and are present in several secretory tissues and hematopoietic cells [11-14].

Rab proteins of both the endocytic and exocytosis pathways play critical roles in cancer progression [15-22]. Recently, Rab27b was reported to regulate invasive growth and metastasis in ER-positive BC cell lines in vitro and in vivo [23]. Also, the presence of Rab27B protein was observed to be associated with a low degree of differentiation and the presence of lymph node metastasis in ER-positive BC. However, this study was limited by its small sample size. Moreover, without adequate patient survival data, we could not investigate the effect of Rab27B on patient prognosis. Thus, the final conclusion in this research did not seem convincing.

Our study was designed to fully investigate Rab27B’s expression pattern in BC as well as its relationship with clinical parameters and survival prognosis. We enrolled 221 BC patients with defined clinicopathologic features to investigate the prognostic value of Rab27B in BC. Our findings strongly suggest that elevated expression of Rab27B may be a risk factor that is predictive of prognosis in BC patients following appropriate therapy.

## Materials and methods

### Patients, tissue specimens and follow up produce

Formalin-fixed, paraffin-embedded tissues (FFPET) of 221 invasive BC samples obtained from 221 women were histologically and clinically diagnosed at the Cancer center, Sun Yat-Sen University (SYSUCC) from January 2000 to December 2002. Additionally, 22 specimens of normal breast tissues from exairesis for non-breast diseases, 40 fibroadenoma of breast (FA), 30 Ductal carcinoma in situ (DCIS) and 40 corresponding metastatic lymph nodes(LNs) were also obtained from the patients in SYSUCC as control. All patients were classified according to the American Joint Committee on Cancer (AJCC) and tumor node metastasis (TNM) classification systems [24]. FFPETs of 221 surgical patients were used to construct the tissue microarray that was used for immunohistochemical (IHC) staining [25,26]. Fourteen 12 Pairs of BC tissues and matched adjacent noncancerous tissues (NAT) were frozen and stored in liquid nitrogen until further use. All samples that we selected were not subjected to preoperative radiotherapy and/or chemotherapy. This study was approved by the Research Ethics Committee of SYSUCC. The patients were followed every 3 months for the first year and then every 6 months for the next 2 years and finally annually. The total disease-specific survival (DSS) follow-up period was defined as the time from operation to the date of cancer-related death or when censured at the lasted date if patients were still alive. All patients still alive at the time of analysis have reached a minimum follow-up period of 60 months (median: 79.0 months; range 60.0 to 112.0 months). A total of 51 patients died from cancer related death during follow-up period.

### Immunohistochemistry staining (IHC)

We used the Dako Real Envision Kit (K5007, Dako) for IHC staining analysis. Hormonal receptors were evaluated with the 1D5 antibody for estrogen receptor a (ERa) and antibody PGR-1A6 for progesterone receptor (PR; Dako). CerbB2 was detected with CB11 (Dako). Only tumor tissues with distinct nuclear staining for ER and PR in >10% of the tumor cells were recorded as positive. Primary antibodies against Rab27B (1:200 dilution, Abcam, USA), E-cadherin, a-Catenin, Fibronectin, Vimentin (1:200 dilution, BD Transduction Laboratories, USA) were used in this study. The staining protocol used in this study was described previously [27].

### Measurements of Rab27B and Epithelial-mesenchymal transition (EMT) markers expression by IHC assay

All sections were stained in DAB for the same amount of time. For each slide, five random fields were selected for scoring, and a mean score of each slide was used in final analyses. Positive staining was accessed by using a five-scale scoring system: 0 (no positive cells), 1 (<25% positive cells), 2 (25%–50% positive cells), 3 (50%–75% positive cells), and 4 (>75% positive cells). To maintain objectivity, we used a four-scale scoring system to describe the intensity of positive staining: 0 (negative staining), 1 (weak staining, light yellow), 2 (moderate staining, yellow brown), and 3 (strong staining, brown). Rab27B expression index = (intensity score) + (positive score). Two independent pathologists without access to the clinicopathologic information performed the scorings. If all scorers assigned consistent results, then the value was selected. When completely different results were given, all of the scorers would work together to confirm a score.

### RNA extraction, reverse transcription and real-time PCR

Total RNA was isolated from 12 pairs of BC tissues and their NAT tissue using TRIZOL reagent (Invitrogen). The extracted RNA was pretreated with RNAase-free DNase, and 2 ug RNA from each sample were used for cDNA synthesis primed with random hexamers. Real-time PCR was carried out using an ABI 7900HT fast real-time system (Applied Biosystems, Foster City, California, USA) to determine the expression pattern of Rab27B mRNA in each of the BC sample as well as the paired NAT tissue. Expression data were normalized to the geometric mean of the housekeeping gene glyceraldehydes 3-phosphate dehydrogenase (GAPDH). The first strand cDNA products were amplified with GAPDH-specific (F: 5’-CCACCCATGGCAAATTCCATGGCA-3’ and R: 5’-TCTAGACGGCAGGTCAGGTCCACC-3’) and Rab27B-specific (F: 5’- TGCGGGACAAGAGCGGTTCCG-3’ and R: 5’- GCCAGTTCCCGAGCTTGCCGTT-3’) primers by PCR.

### Western blot analysis

Equal amounts of BC tissue lysates were resolved by SDS-polyacrylamide gel electrophoresis (SDS-PAGE) and electrotransferred to a polyvinylidene difluoride membrane (Pall Corp., Port Washington, NY). The tissues were then incubated with primary rabbit monoclonal antibodies against Rab27B (1:500 dilution, Abcam, USA). The immunoreactive signals were detected with an enhanced chemiluminescence kit (Amersham Biosciences, Uppsala, Sweden) used in accordance with the manufacturer’s instructions.

### Selection of cutoff score

Receiver operating characteristic (ROC) curve analysis was performed to determine the cutoff score for Rab27B high expression by using the 0,1-criterion [28]. At the Rab27B score, the sensitivity and specificity for each outcome under study was plotted, generating various ROC curves. The score selected as the cutoff value was that which was closest to the point with both maximum sensitivity and specificity. Tumors designated as having low expression of Rab27B were those with scores below or equal to the cutoff value, and tumors of high expression were those with scores above the value. To use ROC curve analysis, the clinicopathological characteristics were dichotomized as follows: tumor size stage (T1 vs. T2 + T3), pathologic grade (I vs. IIIII), lymph node metastasis (absent vs. present), clinical stage (I-II vs. III-IV), and survival status (death due to BC vs. censored).

### Statistical analysis

The statistical significance of the correlation between Rab27B expression level and patient survival was estimated by using the Mantel-Cox log-rank test. ROC curve analysis was conducted to evaluate the predictive value of the parameters. The chi-square test or Fisher’s exact test was used to evaluate the relationship between Rab27B expression and clinicopathological variables. Spearman’s rank correlation test was performed to evaluate the relationship between Rab27B and EMT markers expression by IHC. The multivariate Cox proportional hazards model was used to estimate the hazard ratios and 95% confidence intervals of patient outcome. The relationships between the Rab27B expression levels and disease-specific survival (DSS) were determined by Kaplan–Meier analysis. Log-rank tests were performed to determine the difference in survival probabilities between patient subsets. All *P*-values quoted are two-sided, and *P* < 0.05 was considered to represent a statistically significant result. Statistical analysis was performed by using SPSS 16.0 software (Chicago, IL, USA).

### Consent

Written informed consent was obtained from the patient for publication of this report and any accompanying images.

## Results

### Expression pattern of Rab27B in BC tissues

**Figure 1:**
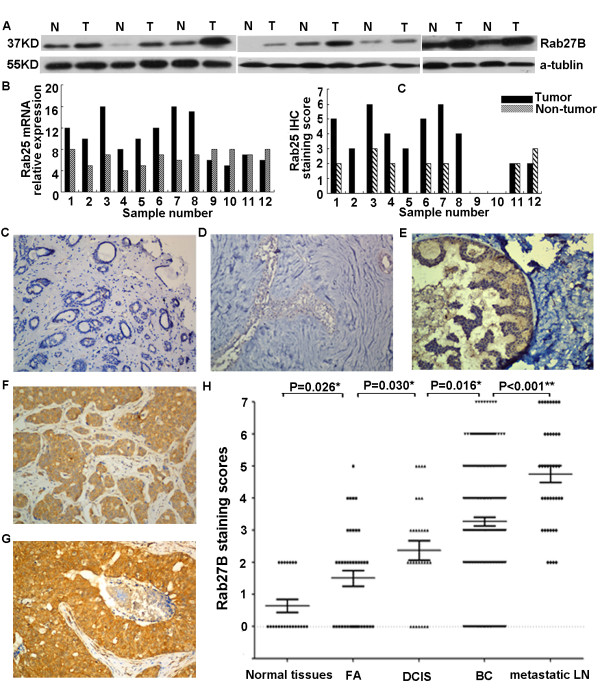
**The expression pattern of Rab27B in BC tissues.** (**A**) Western blot analysis of Rab27B protein expression in 8 representative paired BCs (T) and NATs (N). Equal loading of protein is shown by α-tubulin. (**B**) Rab27B mRNA and protein expression in human BCs (T) and NATs (N) by Real-time PCR (left panel) and IHC analysis (right panel). Rab27B mRNA and protein expression in 12 primary BCs is upregulated compared to their NATs. (**C**) Negative staining of Rab27B in normal breast tissue. (**D**) Weak staining of Rab27B in FA. (**E**) Moderate staining of Rab27B in DCIS. (**F**) BC cells showing strong staining of Rab27B. (**G**) Strong Rab27B staining in metastatic lymph nodes. (**H**) Statistical analysis revealed a significantly ascending pattern of Rab27B expression ranging from NAT to BC to distant metastases nodes.

To investigate the status of Rab27B gene expression in BC, we used Real-time PCR to measure the mRNA expression in 12 pairs of primary BC and NAT specimens. Compared with their NATs, 8 of 12 BC had upregulated expression. Consistently, western blot and IHC analysis showed that the 8 cases also had higher Rab27B protein expression than adjacent tissues. The BC cases with upregulated expression of Rab27B are shown in Figure 1A and 1B. Interestingly, our IHC and Real-time PCR result in these 12 paired BC and NAT specimens demonstrated the protein and mRNA level of Rab27B are positively correlated (r_s_ = 0.705, P < 0.001). IHC analysis showed that positive expression of Rab27B was primarily observed in the cell cytoplasm and membrane. Immunoreactivity of Rab27B protein in BC ranged from 0 to 7. In normal breast tissues and FA tissues, no or weak Rab27B staining was detected (Figure 1C-D). However, Rab27B showed more positive staining in DCIS tissues, BC tissues and metastatic LNs (Figure 1E-1G). An interesting phenomenon was that Rab27B expression appeared to increase incrementally with the magnitude of cancer progression in tissue ( *P* < 0.05. Figure 1H).

### Selection of cutoff score for high expression of Rab27B

The ROC curves for each clinicopathological feature define the cutoff point on the curve closet to the point (0.0, 1.0), which maximized both sensitivity and specificity for the outcome. Tumors with scores above the obtained cutoff value were considered as highly-expressed Rab27B leading to the greatest number of tumors correctly classified as having or not having the clinical outcome. The corresponding area under the curve (AUC) and cutoff scores were collected and are shown in Additional file 1: Figure S1 and Table 1, respectively. In our current study, ROC curve analysis for living status had the shortest distance from the curve to the point (i.e., 0.0, 1.0), and we selected the cutoff value determined by living status. The sensitivity and specificity of Rab27B as a prognosis prediction marker of breast cancer patients are 88.2 and 63.5 respectively. Thus, the cutoff score for high expression of Rab27B was defined when a score greater than 3 was obtained in IHC analysis for Rab27B.

**Table 1:** The corresponding cutoff socre of Rab27B expression for each clinicapathological feature according to ROC curve analysis

**Variable**	**Subvariable**	**Cutoff score**	***P *value**
Histology grade	I(n = 47) vs. IIIII (n = 174)	>3	0.014*
Tumor size stage	T1 (n = 63) vs. T2 + T3 (n = 158)	>2	0.917
Lymph nodes metastasis	absent (n = 105) vs. present (n = 116)	>2	0.064
Clinical stage	I II(n = 181) vs. III (n = 40)	>4	0.046*
Live status	Live (n-171) vs. death (n = 50)	>3	< 0.001*

*Statistically significant difference.

### Association of Rab27B expression with BC clinicopathologic features

According to the cutoff value, high expression of Rab27B was observed in 0 of 22 (0%) normal breast tissues, 5 of 40 (12.5%) FA tissues, 10 of 30 (33.3%) DCIS tissues, 107 of 221 (48.4%) BCs, and 30 of 40 (75.0%) metastatic LNs, respectively (*P* < 0.001, *Chi-square test, Table 2).

**Table 2:** The expression of Rab27B in normal breast tissues and in benign and malignant breast tumors

	**Cases**	**Rab27B expression**		***P *value**
		**Low expression**	**High expression**	
Normal breast tissues	22	22(100%)	0(0%)	
Fibroadenoma of breast	40	35(87.5%)	5(12.5%)	
Ductal carcinoma in situ	30	23(76.7%)	7(23.3%)	
Invasive breast cancer	221	114(51.6%)	107(48.4%)	
Metastatic lymph nodes	40	10(25.0%)	30(75.0%)	<0.001*

*Statistically significant difference.

The rates of high expression of Rab27B in BCs with respect to several standard clinicopathological features are presented in Table 2. High expression of Rab27B was positively correlated with pathology grade, advanced clinical stage, lymph node metastasis, and ER status (*P* < 0.05, Table 1). There was no significant association between Rab27B expression and other clinicopathological features, such as patient age, menopausal status, T stage, RP status, or CerbB2 status (*P* > 0.05, Table 3).

**Table 3:** Relationship between Rab27B expression level and clinicopathologic parameters of BC

**Variable**	**Number of cases**	**Rab27B expression**		***P *value**
		**High expression**	**Low expression**	
**Age** (**years**)				
≥ 47^a^	103	46(44.7%)	57(55.3%)	
< 47	118	61(51.7%)	57(48.3%)	0.297
**Pathologic grade**				
I	47	15(31.9%)	32(69.1%)	
IIIII	174	92(52.9%)	82(47.1%)	0.011*
**Clinical stage**				
III	181	80(44.2%)	101(55.8%)	
III	40	27(67.5%)	13(32.5%)	0.008*
**Tumor size stage**				
T1	63	32(50.8%)	31(49.2%)	
T2 + T3	158	82(51.9%)	76(48.1%)	0.883
**Menopausal status**				
Absent	90	53(58.9%)	37(41.1%)	
Present	131	61(46.6%)	70(53.4%)	0.072
**Lymph node metastasis**				
Metastasis	116	65(56.0%)	51(44.0%)	
No metastasis	105	42(40.0%)	63(60.0%)	0.021*
**ER status**				
Negative	97	37(38.1%)	60(61.9%)	
Positive	124	70(56.5%)	54(43.5%)	0.007*
**PR status**				
Negative	91	39(42.9%)	52(57.1%)	
Positive	130	68(52.3%)	62(47.7%)	0.166
**CerbB2 status**				
0,1+,2+	147	74(50.3%)	73(49.7%)	
3+	74	33(44.6%)	41(55.4%)	0.42
**Living status**				
Live	170	63(37.1%)	107(62.9%)	
Dead	51	44(86.3%)	7(13.7%)	<0.001*

BC breast cancer. a Mean age. *Statistically significant difference.

### Elevated Rab27B expression predicts poor prognosis of BC

**Figure 2:**
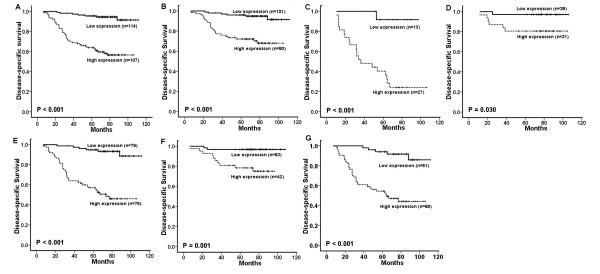
**Influence of Rab27B expression on disease**-**specific survival of BC patients.** (**A**) Kaplan–Meier curves show that patients with elevated Rab27B expression have poor disease-specific survival by analyzing 221 primary BC tissues (*P* < 0.001). Patients with high Rab27B expression have worse disease-specific survival in the stage III group (Panel **B**) and in the stage III group (Panel **C**); Survival time is shorter in patients with high Rab27B expression with T 1 stage (Panel **D**) or T 1+ T 2 stage (Panel **E**); Survival time is shorter in patients with high Rab27B expression whether in the lymph node metastasis group (Panel **F**) and in the no lymph node metastasis group (Panel **G**).

Kaplan–Meier curves showed that, in the primary BC category, the cumulative 5-year disease-specific survival (DSS) rate was 95.6% for patients with lower levels of Rab27B and 64.0% for patients with higher levels of Rab27B expression (*P* < 0.01, Figure 2A). Furthermore, the expression levels of Rab27B were strongly correlated with patients’ survival even after stratifying the patients based upon their clinicopathological variables. Subset analysis showed that high Rab27B expression had a decreased survival time regardless of clinical stage, tumor size stage, and lymph node metastasis (*P* < 0.05, Figure 2B -2G).

### Multivariate survival analysis

Cox regression proportional hazard analyses indicated that high expression of Rab27B was a significant risk factor for adverse DSS (hazard ratio, 8.661; 95% confidence interval [CI], 3.897–19.251, *P* < 0.001). Of the other variables, histology grade, clinical stage, tumor size stage, lymph node metastasis, and PR status were also found to be prognostic predictors of DSS (Table 4). Since clinical stage is a combination of tumor size stage and lymph node involvement, we only include tumor size stage and lymph node metastasis in the multivariate analysis. After multivariate analysis, the expression level of Rab27B was found to be a significant independent prognostic factor of poor DSS in BC patients (hazard ratio, 9.120; 95% CI, 4.056–20.506, *P* < 0.001). Histology grade, tumor size stage, lymph node metastasis, and negative PR status also independently predicted poor overall survival (Table 4).

**Table 4:** Results of univariate and multivariate Cox proportional-hazards analysis for disease-specific survival

**Variable**	**Subvariable**	**All cases (n = 221)**		
		**HR**	**95%CI**	***P***-**value**
**Univariate**				
Age	≥47^a^ yr (n = 103) vs. <47 yr (n = 117)	1.654	0.931-2.938	0.086
Histology grade	I (n = 47) vs. IIIII (n = 174)	7.696	1.871-31.651	0.005*
Clinical stage	I II (n = 181) vs. III (n = 40)	4.059	2.318-7.016	<0.001*
Tumor size stage	T1 (n = 63) vs. T2 + T3 (n = 158)	2.799	1.260-6.219	0.011*
Lymph nodes metastasis	absent (n = 105) vs. present (n = 116)	3.361	1.759-6.422	<0.001*
Menopausal status	absent (n = 131) vs. present (n = 90)	1.549	0.857-2.798	0.147
Rab27B expression	low (n = 107) vs. high (n = 114)	8.661	3.897-19.251	<0.001*
ER status	absent (==97) vs. present (n = 124)	0.588	0.339-1.022	0.06
PR status	absent (n = 91) vs. present (n = 130)	0.505	0.291-0.878	0.015*
CerbB2 status	0, 1+, 2+ (n = 147) vs. 3+ (n = 74)	1.504	0.861-2.628	0.152
**Multivariate**				
Histology grade	I (n = 47) vs. IIIII (n = 174)	4.808	1.154-20.030	0.031*
Tumour size	T1 (n = 63) vs. T2 + T3 (n = 158)	2.630	1.167-5.924	0.020*
Lynph nodes metastasis	absent (n = 105) vs. present (n = 116)	2.696	1.398-5.197	0.003*
Rab27B expression	low (n = 107) vs. high (n = 114)	9.120	4.056-20.506	<0.001*
PR status	absent (n = 91) vs. present (n = 130)	0.412	0.233-0.729	0.002*

^a^ Mean age. Cox propotional hazard regression model, enter; HR, Hazard ratio; CI, confidence interval; *Statistically significant difference.

### Correlation between the expression of Rab27B and EMT markers in BC tissues

**Figure 3:**
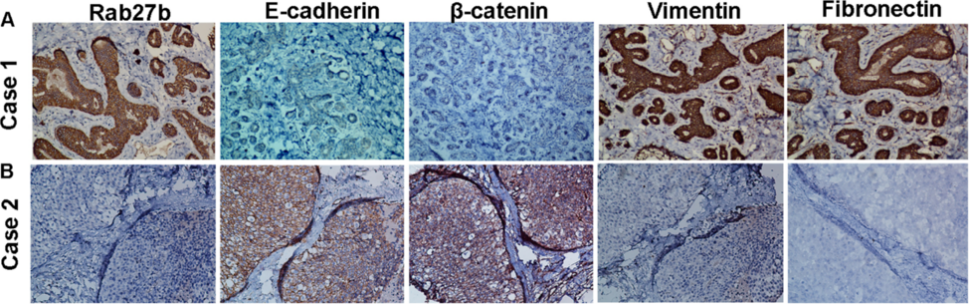
**Correlation between the expression of Rab27B and EMT markers in BC tissues.** (**A**) High expression of Rab27B in BC was accompanied by elevated level of vimentin, fibronectin, and absence of E-cadherin, β-catenin (upper panel). (**B**) Low expression of Rab27B in BC was accompanied by absence of vimentin, fibronectin, and elevated level of E-cadherin, β-catenin (lower panel).

To explore the relationship of Rab27B and EMT process, IHC staining of was performed in 221 primary BC tissues. Representative IHC staining of EMT markers in BC tissues is shown in Figure 3. We observed that Rab27B expression level was positively correlated with the expression level of mesenchymal markers: Vimentin (r_s_ = 0.289, *P* <0.001, n = 221, Spearman’s correlation analysis), Fibronectin (r_s_ = 0.327, *P* < 0.001, n = 221, Spearman’s correlation analysis), and inversely correlated with epithelial markers: E-cadherin (r_s_ = −0.226, *P* = 0.001, n = 221, Spearman’s correlation analysis), β-catenin (r_s_ = −0.389, *P* < 0.001, n = 221, Spearman’s correlation analysis).These data illustrated that overexpression of Rab27B contributed to the EMT process in BC progression.

## Discussion

Rab27B, a regulator of vesicle exocytosis, has been suggested to function as an oncogene in ER-positive breast cell lines both in vitro and in vivo [23]. However, without patients’ survival information, we could not investigate the effect of Rab27B expression on patient prognosis. We performed the present study to investigate the expression dynamics of Rab27B and their clinicopathologic/prognostic significance in BC patients. To the best of our knowledge, this is the first study investigating the expression of Rab27B in a large series of BC patients.

The previous study showed that Rab27B is upregulated in BC [23]; however, Dong et.al reported that the Rab27B expression level was lower in primary hepatocellular carcinoma than in matched adjacent tissues [29]. Our results showed that both the mRNA and protein levels were upregulated in BCs compared with their NATs. Furthermore, we are first to report that expression levels of Rab27B appeared to increase with the magnitude of cancer progression: a significant increasing expression of Rab27B was observed from normal breast tissue, to FA, DCIS, and to invasive BC, metastatic LNs In our study, further correlation analysis revealed for the first time that high Rab27B expression was closely correlated with an aggressive phenotype of BC, including ascending pathologic grade, advanced clinical stage, and lymph node metastasis. These data, in agreement with data of the previous study [23], indicate that increased Rab27B expression corresponds to the progressive magnitude of BC and might facilitate the invasive/metastatic phenotypes of this malignancy, Rab27B is responsible for regulating many secretory mechanisms. Many studies have demonstrated that tumor cells use exosomes to communicate with surrounding tissues and immune cells, creating an immunosuppressive microenvironment for tumor progression [30-33]. Thus, we propose that Rab27B is associated with tumor progression because of its function as a transport vesicle.

It is well known that tumor invasion and metastases are responsible for most cancer-related mortality. As for invasive BC, the lymphatic system is the primary pathway to metastatic disease. For patients with lymph node metastasis, additional chemoradiation is required. Thus, identification of biomarkers that can be used to define the metastatic potential of BC may facilitate the development of appropriate therapeautic strategy earlier in the course of this cancer. In this study, we found that those with higher Rab27B expression are prone to have lymph node metastasis. We recommend that they be identified as patients at high risk in the clinic and that more radical therapy regimens should be delivered to them. Furthermore, the accuracy of predicting lymph node metastasis by examining Rab27B expression level (high vs. low) in our cohort is 60.7% (65/107). In light of these findings, we hypothesize that Rab27B may be a novel predictor of lymph node metastasis in BC patients. To address this issue, a further study in a larger cohort of BC patients is now underway.

As for the underlying mechanism involved in Rab27B-regulating BC invasive and metastasis potential, here we focused on the EMT process. During this process, epithelia cells lose their epithelia adherence, cell-cell contact and their polarity, and undergo remarkable remodeling of the cytoskeleton, all of which facilitate cell invasion and migration [34,35]. Interestingly, our results demonstrated that BCs with Rab27B high expression displayed the enhanced expression of the mesenchymal markers vimentin and fibronectin, and decreased expression of the epithelial markers E-cadherin and β-catenin, suggesting an EMT process during Rab27B-regulating BC development. The acquisition of EMT characteristics may give these BC tumor cells a higher aggressive potential, resulting in the invasive and metastatic behavior. Thus we underscore that the EMT process might be a potential underlying mechanism in Rab27B-regulating BC invasion and metastasis. However, further study is underway to identify the special pathway involved in Rab27B mediated EMT process in BC.

Recently Rab27B was identified as a predictor of prognosis in HCC [29]. With regard to the prognostic effect of Rab27B in BC, our findings show for the first time that BC patients with elevated Rab27B expression had worse survival outcomes than those expressing lower Rab27B, suggesting the clinical value of Rab27B in assessing the prognosis of BC patients. Furthermore, even after by stratified analysis, Rab27B could display a favorable prognosis value in each subgroup. Our findings strongly suggest that Rab27B may be a novel and important prognostic marker for BC patients.

It is noteworthy that the previous study found no Rab27B staining in ER-negative samples [23]; however, we detected positive staining in such samples. The difference may reflect differences in Rab27B status in the samples used in different studies, which obtained tissue samples from different populations. Furthermore, the previous study enrolled only 59 cases, whereas 221 are included in our study, leading to a more convincing result [23]. Furthermore, in our enrolled cases, the prognostic significance of Rab27B expression was not confined to this group. These results confirm that the pro-oncogenic function of Rab27B is not ER-dependent. Thus, Rab27B could be adopted as a widely used biomarker in predicting patients’ survival. However, these results should be confirmed in a large, multicenter trial.

## Conclusion

In summary, our data suggest that increased expression of Rab27B, assocatied with elevated mesenchymal EMT markers, is related to the aggressive and metastatic potential of BC and thus contributes to a poor prognostic phenotype. Examining Rab27B expression by IHC analysis, is an effective way to assess BC patients’ survival outcome. This tool could be of great value to help clinicians to make an optimal individual treatment for BC patients.

## Abbreviations

BC: Breast cancer; FA: Fibroadenoma of breast; DCIS: Ductal carcinoma in situ; LN: Lymph node; NAT: Adjacent noncancerous tissues; IHC: Immunohistochemistry staining; ER: Eestrogen receptor; PR: Progesterone receptor; HER-2: Human epidermal growth factor receptor 2; DSS: Disease-specific survival; ROC: The receiver operating characteristic curve; EMT: Epithelial-mesenchymal transition.

## Competing interests

The authors declare that they have no conflict of interest.

## Authors' contributions

SJM, DX and YXZ carried out and coordinated the study. JXZ, XXH, ZTT, DQ, YJL, HXD, MBC, DZL and MYH performed the experiments. JXZ and XXH analyzed the data. JXZ wrote the paper. All authors read and approved the final manuscript.

## Supplementary Material

Additional file 1 ** Figure S1.**Receiver operating characteristic curve analysis was used to determine the cutoff score for the high expression of Rab27B. The sensitivity and specificity scores of each outcome were plotted: (A) Histology grade (*P* = 0.014) (B) Tumor size stage (*P* = 0.917); (C) Lymph node metastasis status (*P* = 0.064); (D) Clinical stage (*P* = 0.046); (E) Survival status (*P* < 0.001). Click here for file
