# Supplementary figures and images for: Overexpression of the secretory small GTPase Rab27B in human breast cancer correlates closely with lymph node metastasis and predicts poor prognosis
Source: J Transl Med. 2012 Dec 5;10:242. doi: 10.1186/1479-5876-10-242 (PMC3539959; doi:10.1186/1479-5876-10-242)

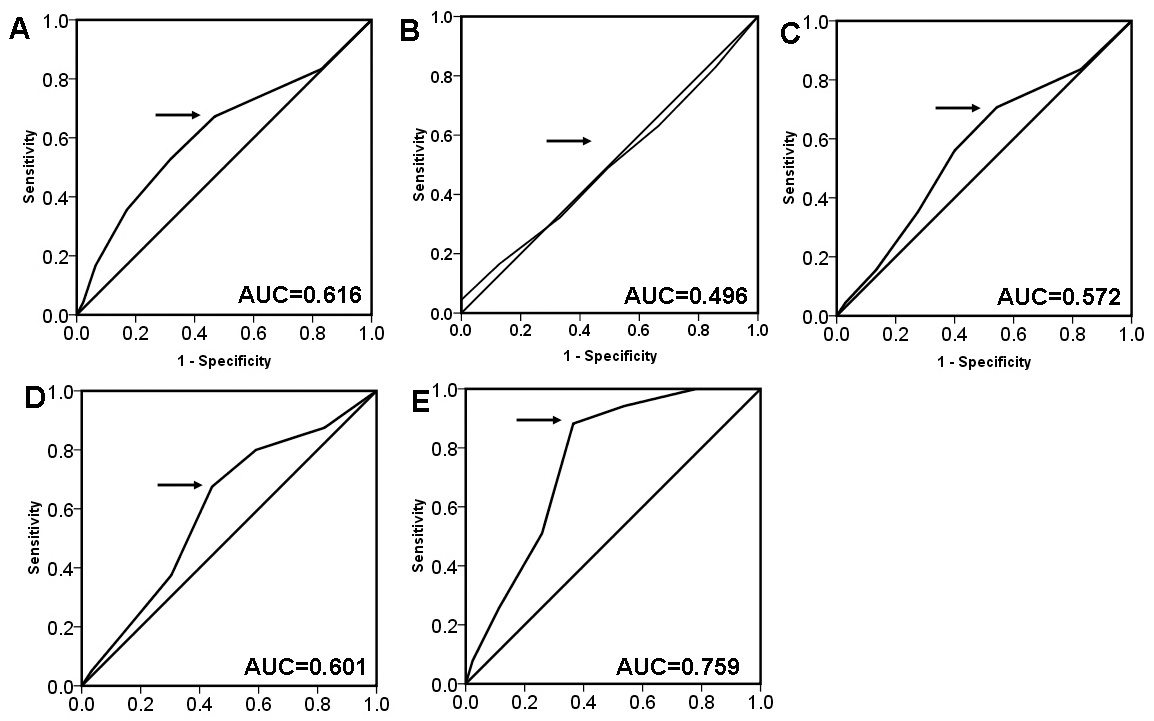

Supplement: Additional file 1 — Figure S1.Receiver operating characteristic curve analysis was used to determine the cutoff score for the high expression of Rab27B. The sensitivity and specificity scores of each outcome were plotted: (A) Histology grade (P = 0.014) (B) Tumor size stage (P = 0.917); (C) Lymph node metastasis status (P = 0.064); (D) Clinical stage (P = 0.046); (E) Survival status (P < 0.001). [file 1479-5876-10-242-S1.jpeg]
